# RE-ENGAGING AFRICAN AMERICAN AND BLACK OLDER ADULTS DURING THE COVID-19 ERA: A FOCUS GROUP STUDY

**DOI:** 10.1093/geroni/igae098.0106

**Published:** 2024-12-31

**Authors:** Rupal Parekh, Kelsi Carolan, Georgia Powell, Christine Tocchi

**Affiliations:** University of Connecticut, New York, Connecticut, United States; University of Connecticut, Storrs, Connecticut, United States; Powell Consulting, Windsor, Connecticut, United States; University of Connecticut, Storrs, Connecticut, United States

## Abstract

Churches and senior centers are social engagement hubs for older Black and African Americans (BAA) that can positively impact well-being. Although these facilities re-opened after the COVID-19 pandemic, engagement with these facilities has not returned to pre-pandemic levels. Our long-term goal is to develop a behavioral intervention to address the re-engagement needs/concerns of BAA older adults as a means of preventing/reducing depressive symptoms and disability. In the first phase of this work, we used a community-based participatory research approach to determine barriers to, and facilitators of, activity reengagement through the perspectives of leaders of BAA churches and senior centers around Hartford, Connecticut. We conducted four focus groups with key stakeholders (n=4 senior center staff; n=4 senior, center staff; n=5 church staff; n=4 church staff) between May 2023 and July 2023. Using a Framework Method approach participants were asked to define engagement as it relates to senior center and church involvement since re-opening and to quantify, in their own words, the problem of disengagement. Participants were also asked about the effectiveness of online engagement vs. in-person engagement and members experience(s) with social isolation and loneliness. Line-by-line thematic analysis of the transcriptions was used to identify and group meaningful phrases and responses into major themes. Three organizing themes emerged: “transportation” concerns impacting members during COVID-19 pandemic, increased health issues among members; and issues and concerns related to family support. Leaders from churches and senior centers provided valuable insights into the overarching issues affecting re-engagement among older BAA senior center and church members.

